# Modeling method and verification of interface contact stiffness based on micro-surface morphology detection

**DOI:** 10.1371/journal.pone.0345403

**Published:** 2026-04-21

**Authors:** Shikun Lu, Bokai Feng, Yunshuai Chen

**Affiliations:** 1 School of Mechanical and Electrical Engineering, Heze University, Heze, China; 2 School of industrial software of Henan University of Engineering, Zhengzhou, China; Koneru Lakshmaiah Education Foundation / Indian and Xidian University, INDIA

## Abstract

A novel elastic-plastic theoretical calculation model for interfacial contact stiffness has been deduced and established based on the observed characteristics of the interface microstructure. In this research, the deformation of asperities under the action of loads is partitioned into two stages. In light of the feature that the interfacial plastic deformation can be eliminated after multiple contact compressions, a computational model for interfacial contact characteristics is put forward, which solely takes into account the elastic deformation of asperities. Through the numerical simulation of the constructed model, the nonlinear relationships between the interfacial contact loads, contact stiffness, and interfacial contact distance are intuitively revealed. The effectiveness of the proposed model is verified by comparing it with experimental results, Xiao Huifang’s corrected experimental contact model, the KE statistical contact model, and the GW model. The proposed calculation model of rough interfacial contact stiffness offers certain references for the calculation of contact loads and contact stiffness, as well as for the performance prediction and optimization of mechanical interfaces.

## 1. Introduction

Interface contacts are indispensable in mechanical devices with different mechanical parts assembled together and they play an important role in moment transmission of devices, transmission of movement and energy consumption. Contact stiffness is an important parameter to characterize the tribological interfaces and has important influences on overall static and dynamic characteristics (e.g., overall stiffness and modal frequency) of the assembled system [1–4]. Studying contact stiffness of part interfaces [1,3–7] is of important significance to master mechanical properties of devices and improve precision of devices [8]. The interface contact behaviors of high-end devices have obvious influences on performance of devices. It has been proved that interface stiffness influences stiffness of machine tools greatly [9]. In the machine tool system, insufficient interfacial contact stiffness of a single key part may affect stiffness of the whole system. From the microscopic significance, part surfaces in engineering are rough and unsmooth [10]. Interface stiffness is attributed to concavities and convexities on the rough interface [1,4,11]. Contact stiffness can be used to describe contact states of interfaces in the mechanical system [12]. Interfacial contact stiffness mainly includes static normal stiffness, static tangential stiffness [13], dynamic normal stiffness, and dynamic tangential stiffness. These characteristics are related to microstructure of interface surface, physical and chemical properties of surface layer as well as surface processing method. Additionally, surface processing and lubrication method also can influence contact characteristics of interfaces [14]. To master influencing factors of interfacial contact stiffness and disclose the involved parameter relations, the variation laws of interfacial contact stiffness are studied and influences of surface processing methods and roughness are investigated [15]. As a result, contact stiffness of some interfaces which are difficult to be measured is gained. In a word, it is very necessary for researchers to study interfacial contact stiffness.

Based on the contact fractal theory and micro-contact size distribution functions, Wen Shuhua et al. [16] constructed a fractal model of normal interfacial contact stiffness involving influences of domain extension factor of micro-contact size distribution. Zhang Xueliang et al. [17,18] constructed a fractal model of static basic characteristic parameters of machine interfaces. They also studied fractal model of normal contact stiffness of rough surfaces, and disclosed influences of interface parameters on interface performances. Jiang et al. [19] built the contact stiffness model of basic planar contact based on fractal theory.You Jinmin and Chen Tianyu [20] deduced the fractal model of mechanical interface normal contact parameters based on fractal geometric theory and contact mechanics theory. Moreover, they determined the normal contact stiffness of interfaces under the known surface material properties, normal forces and surface fractal parameters. Wilson et al. [21] analyzed elastic-plastic contact problems of rough surfaces and constructed the contact fractal model based on Hertz contact theory. Greenwood and Williamson [22] built an elastic contact model which elaborates how contact deformation depends on surface morphology by combining with early surface contact theory. Based on GW model, Chang et al. [23] proposed an elastic-plastic model to analyze rough interface contacts. Zhao et al. [24] constructed a model of asperity contact deformation from elastic deformation to complete plastic flow, and developed an elastic-plastic contact model between two rough surfaces. Kogut and Etsion [25] built an elastic-plastic finite element model of friction-free contact between rigid plate and deformable spheres under loads, and disclosed influences of interface parameters on interface performances. Jackson and Green [26] carried out a finite element study on elastic-plastic semispherical contact problem and standardized research results to be applicable to macroscopic and microscopic contacts. Brake [27] proposed a new normal elastic-plastic contact model based on material properties. This model covered elastic behaviors of interface contact, mixed elastic-plastic behaviors and full plastic flow behaviors. Chen and Etsion [28] combined existing single-coating rough contact model into the GW-based multi-coating roughness surface model and proposed an elastoplasticity coating rough surface contact model. Sepehri [29] built a finite element elastoplasticity model of rough surface contact, and carried out finite element analysis of rough surface contact. You Jinmin and Chen Tianyu [30] also constructed calculation models of contact area, contact load and contact stiffness of mechanical interfaces. This model gave comprehensive considerations to elastic deformation, elastic-plastic deformation and plastic deformation of asperities based on statistical theory, fractal theory and contact mechanics theory. Jackson and Streator [31] used contact area as the function of contact load for prediction and built a multi-scale model of rough surface by integrating influences of multi-scale rough surface deformation into a simple framework. Wang et al. [32] refined and smoothed rough surface by spectral interpolation method to simulate contact and studied micro elastic-plastic contact between rough surface and rigid plate under normal and tangential loads through a finite element method. Zhou et al. [33] carried out multi-scale modeling and finite element analysis of dynamic characteristic of bolt connected composite structures, and built a stick-slip friction model with a single degree of freedom (SDOF). This model took micro-scale contact surface effect into account. Sun et al. [34] proposed a semi-analytical model to calculate the tangential-to-normal stiffness ratio of static contact among rough surfaces. According to results of numerical simulation, Xiao and Sun [35] established an explicit approximate expression of contact stiffness under normal loads. Kucharski and Starzyński [36] studied contact characteristics of deformable random rough surface and rigid smooth surface from experimental and theoretical aspects. Interfacial contact characteristics based on theoretical modeling technique are studied through contact mechanics theory, statistical theory and fractal geometric theory. A microscopic contact model of different rough surfaces was built, which was applicable to calculate contact stiffness, contact damping and other parameters. Typical models include the GW model, the ZMC model, etc. Interfacial asperities shapes are related to processing cutters, processing textures and physical and chemical properties of materials. Therefore, existing theoretical calculation models have great errors with practical results due to the hypotheses of asperity shapes. Hence, it is necessary to further study and perfect the calculation model of interfacial contact stiffness to provide richer and more-dimensional research explorations.

Based on morphological characteristics of interfacial asperity, a new calculation model of interfacial contact stiffness is established and the effectiveness of the model calculation is verified by an experiment. As a result, contact load and contact stiffness of interfaces are acquired. Moreover, influences of standard deviation of different average asperity heights on contact load and contact stiffness are taken into account. In this study, a new calculation method of interfacial contact stiffness is proposed.

## 2. Normal contact theoretical model of rough interfaces

**Fig 1:**
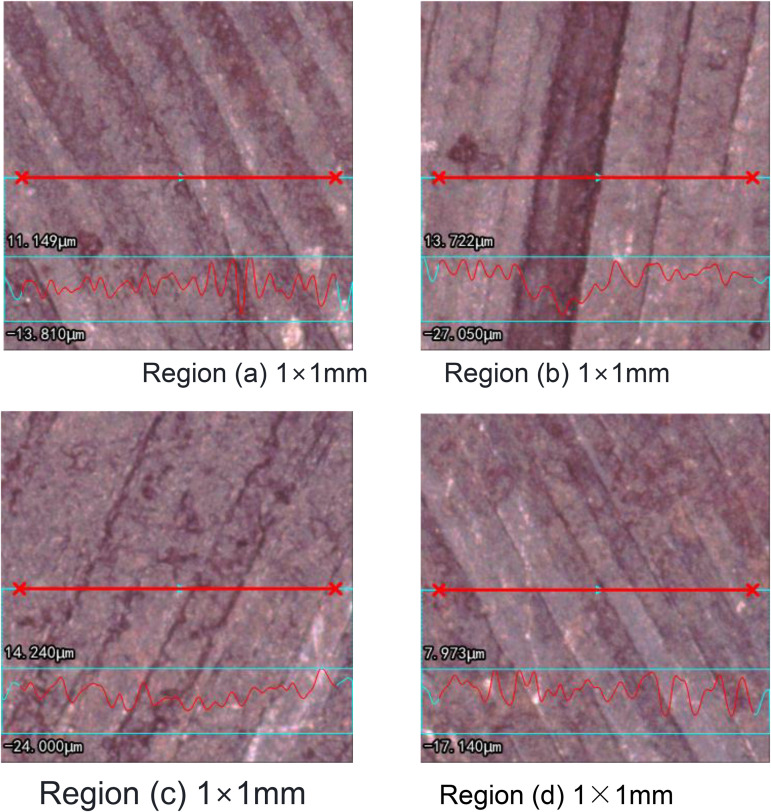
Observation results of the electron microscope interface.

**Fig 2:**
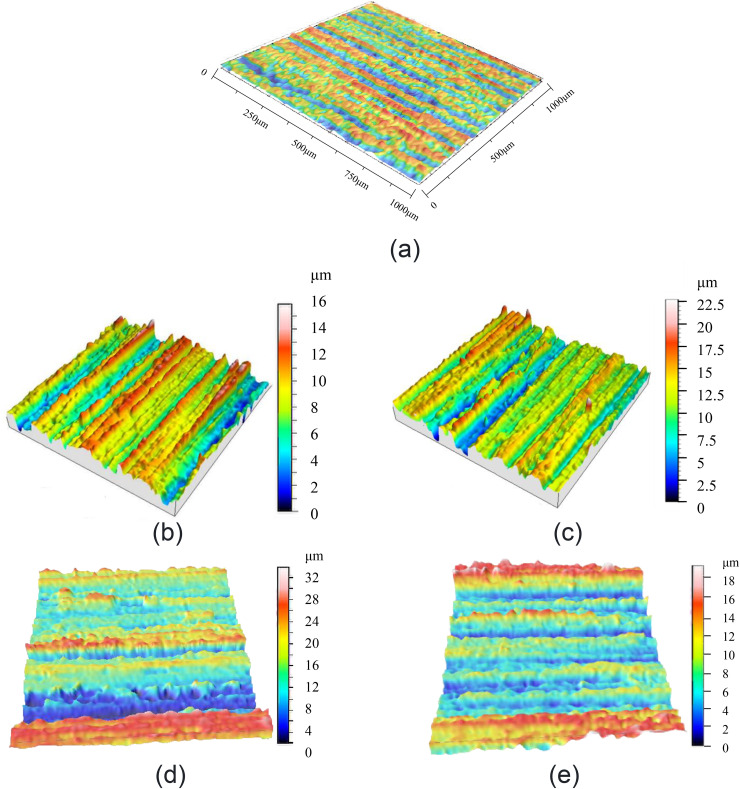
Three-dimensional morphological characteristics of the interface surface. **(a)** Surface contour measurement diagram 1. **(b)** Surface contour measurement diagram 2. **(c)** Surface contour measurement diagram 3. **(d)** Surface contour measurement diagram 4.

The static and dynamic contact characteristics of the microscopically rough interface are determined by the contact state of the micro-asperities at the micro-scale of the two surfaces. Fig 1 shows the actual machined surface measured by an electron microscope. It can be seen that the surface has a certain texture direction, and the distribution characteristics of the transverse and longitudinal micro-asperities are significantly different. The micro-asperities formed along the grain direction are often strip-shaped protrusions and valleys [37] (as shown in Fig 2, the result was obtained through detection by a white light interference confocal microscope). Based on this, establish an interfacial contact model.

Since steel is a kind of elastoplastic material, there’s no explicit boundary among deformation stages during compressed deformation of asperities. Elastic deformation and plastic deformation often exist in the deformation process at the same time. There’s plastic deformation in elastic deformation and some elastic deformation in plastic deformation. There are different types of dominant deformation during different deformation processes. According to this phenomenon, the contact deformation of cylindrical asperity is divided into two stages. Elastic deformation takes the dominant role in the first stage, but plastic deformation takes the dominant role in the second stage.

**Fig 3:**
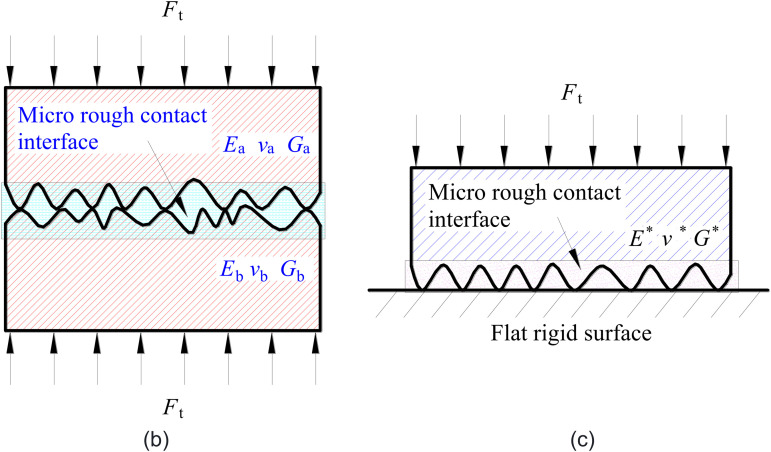
Contact schemes of rough surfaces. **(a)** Contact between two rough surfaces. **(b)** Surface equivalent contact (Contact between a rigid surface and a rough surface).

The contact problem of two rough surfaces under normal static loads is studied and a calculation model of interfacial contact stiffness is built based on the hypothesis that heights of all asperities on the interface obey a normal distribution. A load analysis is carried out on the contact behaviors of rough interfaces. Later, the normal deformation of asperities and the loads produced in the first and second stages of deformation are investigated. Based on statistical theory, the normal contact stiffness, contact area and contact load of the whole interface are acquired. The contact of rough interfaces is shown in Fig 3(a). In Fig 3(a), the Young’s moduli are *E*_a_ and *E*_b_, and the Poisson’s ratios are *v*_a_ and *v*_b_ respectively. The contact between the two interfaces can be equivalent to the contact between an asperity and a rigid surface, as shown in Fig 3(b).

### 2.1 Contact analysis of asperities

**Fig 4:**
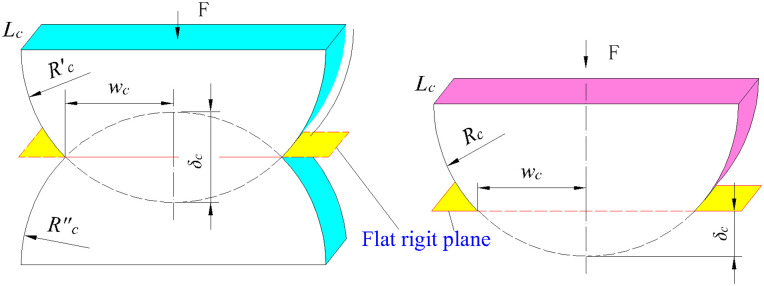
Contact of two asperities and contact between asperity and rigid surface. **(a)** Contact of two asperities. **(b)** Contact between asperity and rigid surface.

The positive contact between two interfacial asperities (Fig 4(a)) can be equivalent to the contact between asperities and a rigid surface (Fig 4(b)). Under the action of positive loads, the interfacial asperities undergo the first deformation stage. With the gradual increase of loads, more plastic deformation develops and it enters into the second deformation stage [19].

In the elastic deformation stage, the relationship between contact load and the concave-convex semi-width (*w*_c_) is [38]:


$$\overset{s}{\underset{e}{f}}\left(w_{c}\right)\text{=}\frac{\pi E^{*}\overset{\text{2}}{\underset{c}{w}}L_{c}}{4R_{c}}$$ (1)

where E^*^ is the equivalent elastic modulus of interface contact materials. It meets $1/E^{*}=\left(1-\overset{\text{2}}{\underset{a}{v}}\right)/E_{a}+\left(1-\overset{\text{2}}{\underset{b}{v}}\right)/E_{b}$, where *E*_a_ and *E*_b_ are elastic modulus of materials of two asperities. *v*_a_ and *v*_b_ are Poisson’s ratio of asperity A and asperity B, respectively. If two asperities have the same material, E^*^ meets $1/E^{*}=2\left(1-\overset{\text{2}}{\underset{c}{v}}\right)/E_{c}$, where *E*_c_ is the elasticity modulus of materials and *v*_c_ is Poisson’s ratio of materials. *L*_c_ is the length of cylindrical surface and *R*_c_ is the radius of the cylindrical surface.

According to geometric relations of normal deformation, cylindrical radius and contact semi-width, the virtual contact width can be expressed as:


$${\overset{\text{2}}{\underset{c}{w}}}^{\prime }\text{=}2R_{c}\delta_{c}-\overset{\text{2}}{\underset{c}{\delta }}$$ (2)

where *δ*_*c*_ is the normal deformation of cylindrical asperities.

Substitute Eq. (2) into Eq. (1) and the force at virtual contact points can be gained:


$${\overset{s}{\underset{e}{f}}}^{\prime }\left(\delta_{c}\right)\text{=}\frac{\pi }{4R_{c}}E^{*}\left(2R_{c}\delta_{c}-\overset{\text{2}}{\underset{c}{\delta }}\right)L_{c}$$ (3)

It can be seen from References [39,40] that the implicit expression of loads produced in the elastic normal deformation stage of asperity is:


$$\overset{s}{\underset{e}{f}}\left(\delta_{c}\right)=\frac{\pi \delta_{c}E^{*}L_{c}}{ln\left\{4\pi E^{*}R_{c}L_{c}/\overset{s}{\underset{e}{f}}\left(\delta_{c}\right)\right\}-1}$$ (4)

The explicit expression of contact load to contact deformation is:


$$\overset{s}{\underset{e}{f}}\left(\delta_{c}\right)\text{=}\frac{m_{ci}\pi }{4R_{c}}E^{*}\left(2R_{c}\delta_{c}-\overset{\text{2}}{\underset{c}{\delta }}\right)L_{c},\text{\textbackslash{}hspace\{0.17em}\;\;\;\;\}0<\delta_{c}\leq \overset{s}{\underset{epc}{\delta }}$$ (5)

where *S*_y_ is the yield strength of materials and it is determined 355MPa. $\overset{s}{\underset{epc}{\delta }}$ can be obtained from Eq. (8). The tangent modulus (*E*_t_) is 2.06 × 1010 Pa. The elasticity modulus (*E*_c_) is 2.07 × 10^11^Pa. Poisson’s ratio of materials is 0.286. The equivalent radius (*R*_c_) of asperity is 0.3 mm. The length of cylindrical surface (*L*_c_) is 0.6 mm. Based on calculation results of implicit expression, numerical fitting is carried out to explicit expression. On this basis, the coefficient (*m*_ci_) is calculated 0.16. Therefore, the explicit expression of contact load produced by the elastic deformation stage of asperity is:


$$\overset{s}{\underset{e}{f}}\left(\delta_{c}\right)\text{=}\frac{\text{1}}{25R_{c}}\pi E^{*}\left(2R_{c}\delta_{c}-\overset{\text{2}}{\underset{c}{\delta }}\right)L_{c}$$ (6)

It is necessary to verify validity of explicit expression of contact loads. This expression is verified by asperity radius, cylindrical contact length of asperity and yield strength of materials.

By comparing the calculation results of single – cylindrical – surface contact under different cylindrical surface radii, it is found that in the elastic deformation stage of cylindrical surface contact, when the radius span reaches 1000 times, the contact loads calculated by the two models are extremely close. Moreover, the greater the degree of deformation, the smaller the error. Through analyzing the calculation results of single cylindrical surface contact under different cylindrical surface lengths, it can be seen that in the elastic deformation stage, when the span of the cylindrical – surface length is 40 times, the contact loads calculated by the two models are also very close. In addition, the comparison of the calculation results of single cylindrical surface contact with different material yield strengths indicates that in the elastic deformation stage, the maximum error of the contact loads calculated by the two models is less than 10%, and in most cases, the error is less than 5%. When the ratio of the elastic modulus to the yield strength (E/Sy) fluctuates by 30% around 580, the error is even lower. In conclusion, the above – mentioned research demonstrates the effectiveness of the explicit calculation model in calculating contact loads during the elastic contact deformation stage.

This paper uses equation (6) as the calculation formula for the contact load generated during the first deformation stage of the asperity. Take the interval $0<\delta_{c}\leq 7\overset{s}{\underset{epc}{\delta }}$ as the deformation interval of the first deformation stage of the asperity.

The expression of transition load produced during transition from the first deformation stage to the second deformation stage is [41]:


$$\overset{s}{\underset{ep}{f}}=7\overset{s}{\underset{epc}{f}}=7\pi R_{c}{\left(CS_{y}\right)}^{\text{2}}\frac{L_{c}}{E^{*}}$$ (7)

where $\overset{s}{\underset{epc}{f}}$ is the loads produced by deformation points when the theoretical elastic stage enters into the elastic-plastic stage [41]. *C* expresses the ratio between maximum contact pressure and maximum stress: *C = p*_o_*/σ*_e-max_. *S*_y_ is the yield strength of materials. *S*_y_ is defined as equal to *σ*_e-max_. *C* is also equal to *p*_*o*_*/S*_*y*_. When Poisson’s ratio *v*_*c*_ ≤ 0.1938, *C* = 1/[1 + 4(*v*_*c*_-1)*v*_*c*_]^0.5^. When Poisson’s ratio *v*_*c*_ ≤ 0.1938, *C* = 1.164 + 2.975*v*_*c*_ *−*2.906*v*_*c*_^2^. *L*_c_ refers to the length of a column and E^*^ is the equivalent elasticity modulus of asperity contact.

The transition deformation from the first deformation stage to the second deformation stage is expressed as [41]:


$$\overset{s}{\underset{ep}{\delta }}=7\overset{s}{\underset{epc}{\delta }}=7R_{c}{\left(\frac{S_{y}}{E^{*}}\right)}^{\text{2}}\left[2ln\left(\frac{2E^{*}}{S_{y}}\right)-1\right]$$ (8)

where $\overset{s}{\underset{epc}{\delta }}$ refers to deformation at the deformation points when the theoretical elastic stage enters into the elastic-plastic stage [41].

In the first deformation stage, given the contact load, the transverse semi-contact width of asperity [40] can be expressed as:


$$w_{c}\text{=7}{\left\{4\overset{s}{\underset{e}{f}}R_{c}/\left(\pi L_{c}E^{*}\right)\right\}}^{0.5}$$ (9)

According to Eq. (9) and definition of area, the contact area in the first deformation stage of asperity can be gained:


$$\overset{s}{\underset{ci\_e}{s}}\left(\delta_{c}\right)=2{\left[4\overset{s}{\underset{epc}{f}}R_{c}/\left(\pi L_{c}E^{*}\right)\right]}^{0.5}L_{c}$$ (10)

$\overset{s}{\underset{epc}{f}}$ is substituted into Eq. (10) and it can get:


$$\overset{s}{\underset{ci\_e}{s}}\left(\delta_{c}\right)=0.8{\left(2R_{c}\delta_{c}-\overset{\text{2}}{\underset{c}{\delta }}\right)}^{0.5}L_{c},\text{\textbackslash{}hspace\{0.17em}\;\;\;\;\}0<\delta_{c}\leq \overset{s}{\underset{ep}{\delta }}$$ (11)

Eq. (11) is the calculation formula for the contact area of a single asperity in the first deformation stage.

An asperity enters the second deformation stage upon compression. In this stage, the force produced by the deformation of the asperity in the second deformation stage is related to the contact area.

**Fig 5:**
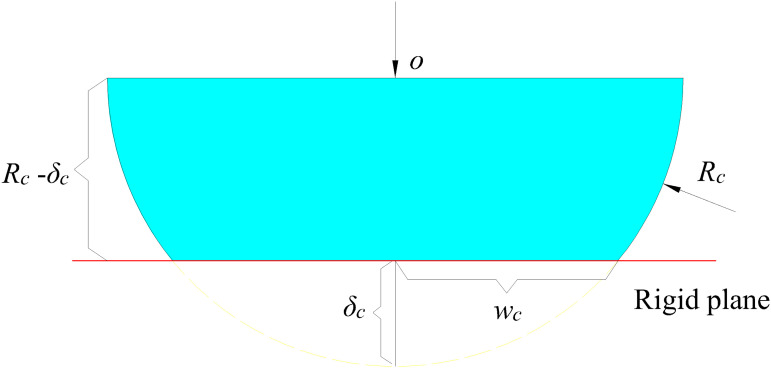
Contact between column and rigid plane.


The contact area of the asperity in the second deformation stage can be calculated according to the geometric relationship. It is approximately equal to the product of the asperity’s contact width and contact length. According to Fig 5, the contact area can be expressed as:

$$\overset{s}{\underset{ci\_p}{s}}\left(\delta_{c}\right)=2L_{c}{\delta_{c}}^{\frac{\text{1}}{\text{2}}}{\left(2R_{c}-\delta_{c}\right)}^{\frac{\text{1}}{\text{2}}}$$ (12)

It can be seen from Reference [38] that the force produced in the plastic deformation stage of asperity can be expressed as the multiplication of material hardness *H*_0_ and contact area of asperity.


$$\overset{s}{\underset{p}{f}}\left(\delta_{c}\right)={\text{2}}^{\frac{\text{3}}{\text{2}}}H_{\text{0}}L_{\text{ci}}{\left(R_{c}-\frac{\delta_{c}}{\text{2}}\right)}^{\frac{\text{1}}{\text{2}}}{\delta_{c}}^{\frac{\text{1}}{\text{2}}}$$ (13)

where *f*_p_ refers to the contact force between spherical asperity and rigid plane and the independent variable *δ*_c_ meets the relation that $\delta_{\text{c}}\geq \overset{s}{\underset{\text{ep}}{\delta }}$.

### 2.2 Normal contact characteristics of interfaces

**Fig 6:**
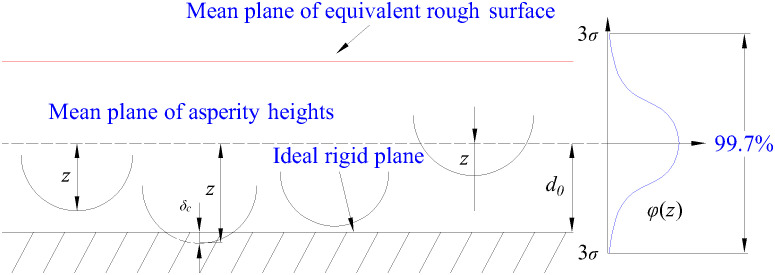
Contact of equivalent rough surface and rigid surface from the microscopic perspective.


It can be seen from Fig 6 that height distribution of asperities on rough surface meets the normal distribution and the probability density function of normal distribution is *φ* (*z*).

$$\varphi \left(z\right)=\sigma^{-1}{\left(2\pi \right)}^{-1/2}exp\left[-z^{\text{2}}/\left(2\sigma^{\text{2}}\right)\right]$$ (14)

where *σ* is the standard deviation of average heights of asperity (root-mean-square) and it can be calculated according to Eq. (15).


$$\sigma^{\text{2}}=\frac{\text{1}}{L}{\int_{\text{0}}^{L_{q}}z\left(x\right)}^{\text{2}}dx$$ (15)

The relationship between *σ* and *R*_a_ is determined by properties of surface to some extent. For the plane conforming to Gaussian (normal) distribution, there’s [38] $\sigma ={\left(\pi /2\right)}^{1/2}R_{\text{a}}$.

According to variable relations in Fig 6, there’s:


$$\delta_{\text{C}}=z-d$$ (16)

The following hypotheses are made: (a) transverse distribution of asperities on surface is random; (b) all asperities have the same radius of curvature [38]; (c) the peak heights of asperities conform to normal distribution; (d) there’s enough distance among asperities; (e) There’s no mutual influence among deformations of asperities. The total number of asperities per unit transverse length is *N*_z_. Then, the number of bulges perpendicular to the transverse width of interface texture is *N*_z_*L*_tr_. The probability of asperity within the height range of [*z*, *z + dz*] is *N*_z_*L*_tr_*φ(z)dz*.

Under the action of loads, multiple asperities on the interface enter into the first and second deformation stages successively. The total loads produced by the interface are the sum of loads produced by all deformed asperities. Combining the loads of asperities and their distribution along height, including asperities of the first and second deformation stages, the total contact loads of the interface can be expressed as:


$$\begin{array}{c} \overset{jo}{\underset{t}{F}}\left(\delta_{c}\right)\text{=}N_{z}L_{tr}\int_{\text{0}}^{d_{\text{0}}+7\overset{s}{\underset{epc}{\delta }}}\overset{s}{\underset{e}{f}}\left(\delta_{c}\right)\cdot \varphi \left(z\right)dz\text{+}N_{z}L_{tr}\int_{d_{\text{0}}+7\overset{s}{\underset{epc}{\delta }}}^{\infty }\overset{s}{\underset{p}{f}}\left(\delta_{c}\right)\cdot \varphi \left(z\right)dz\mathrm{\backslash hfill}\\ =N_{z}L_{tr}\int_{\text{0}}^{d_{\text{0}}+7\overset{s}{\underset{epc}{\delta }}}\frac{\text{1}}{25R_{c}}\pi E^{*}\left(2R_{c}\delta_{c}-\overset{\text{2}}{\underset{c}{\delta }}\right)L_{c}\cdot \sigma^{-1}{\left(2\pi \right)}^{-1/2}exp\left(-z^{\text{2}}/2\sigma^{\text{2}}\right)dz\mathrm{\backslash hfill}\\ +N_{z}L_{tr}\int_{d_{\text{0}}+7\overset{s}{\underset{epc}{\delta }}}^{\infty }{\text{2}}^{\frac{\text{3}}{\text{2}}}H_{\text{0}}L_{\text{ci}}{\left(R_{c}-\frac{\delta_{c}}{\text{2}}\right)}^{\frac{\text{1}}{\text{2}}}{\delta_{c}}^{\frac{\text{1}}{\text{2}}}\cdot \sigma^{-1}{\left(2\pi \right)}^{-1/2}exp\left(-z^{\text{2}}/2\sigma^{\text{2}}\right)dz\mathrm{\backslash hfill} \end{array}$$ (17)

According to variable relations in Fig 6, there’s *δ*_c_ = *z* - *d*_0_, which is substituted into Eq. (17), as a result, the interfacial contact force of the variable *d*_0_ can be gained:


$$\begin{array}{c} \overset{jo}{\underset{t}{F}}\left(\delta_{c}\right)\text{=}N_{z}L_{tr}\int_{\text{0}}^{d_{\text{0}}+7\overset{s}{\underset{epc}{\delta }}}\overset{s}{\underset{e}{f}}\left(z-d_{\text{0}}\right)\cdot \varphi \left(z\right)dz\text{+}N_{z}L_{tr}\int_{d_{\text{0}}+7\overset{s}{\underset{epc}{\delta }}}^{\infty }\overset{s}{\underset{p}{f}}\left(z-d_{\text{0}}\right)\cdot \varphi \left(z\right)dz\mathrm{\backslash hfill}\\ =N_{z}L_{tr}\int_{\text{0}}^{d_{\text{0}}+7\overset{s}{\underset{epc}{\delta }}}\begin{array}{c} \frac{\text{1}}{25R_{c}}\pi E^{*}\left\{\begin{array}{c} @l@2R_{c}\left(z-d_{\text{0}}\right)\\ -{\left(z-d_{\text{0}}\right)}^{\text{2}} \end{array}\right\}L_{c}\\ \cdot \sigma^{-1}{\left(2\pi \right)}^{-1/2}exp\left\{-z^{\text{2}}/\left(2\sigma^{\text{2}}\right)\right\}dz \end{array}\mathrm{\backslash hfill}\\ +N_{z}L_{tr}\int_{d_{\text{0}}+7\overset{s}{\underset{epc}{\delta }}}^{\infty }\begin{array}{c} {\text{2}}^{\frac{\text{3}}{\text{2}}}H_{\text{0}}L_{\text{ci}}{\left(R_{c}-\frac{z-d_{\text{0}}}{\text{2}}\right)}^{\frac{\text{1}}{\text{2}}}{\left(z-d_{\text{0}}\right)}^{\frac{\text{1}}{\text{2}}}\\ \cdot \sigma^{-1}{\left(2\pi \right)}^{-1/2}exp\left\{-z^{\text{2}}/\left(2\sigma^{\text{2}}\right)\right\}dz \end{array}\mathrm{\backslash hfill} \end{array}$$ (18)

The total contact area of the interfaces is obtained through the integral of the contact areas of all asperities. It is the sum of the contact areas of asperities that are deformed in the first and second stages. The total contact area of the interfaces is:


$$\begin{array}{c} \overset{jo}{\underset{t}{A}}\left(\delta_{c}\right)=N_{z}L_{tr}\int_{d_{\text{0}}}^{d_{\text{0}}+7\overset{s}{\underset{epc}{\delta }}}\overset{s}{\underset{ci\_e}{s}}\left(z-d_{\text{0}}\right)\cdot \varphi \left(z\right)dz\mathrm{\backslash hfill}\\ +N_{z}L_{tr}\int_{d_{\text{0}}+7\overset{s}{\underset{epc}{\delta }}}^{\infty }\overset{s}{\underset{ci\_p}{s}}\left(z-d_{\text{0}}\right)\cdot \varphi \left(z\right)dz\mathrm{\backslash hfill}\\ =N_{z}L_{tr}\int_{d_{\text{0}}}^{d_{\text{0}}+7\overset{s}{\underset{epc}{\delta }}}\begin{array}{c} 0.8{\left\{2R_{c}\left(z-d_{\text{0}}\right)-{\left(z-d_{\text{0}}\right)}^{\text{2}}\right\}}^{0.5}L_{c}\\ \cdot \sigma^{-1}{\left(2\pi \right)}^{-1/2}exp\left\{-z^{\text{2}}/\left(2\sigma^{\text{2}}\right)\right\}dz \end{array}\mathrm{\backslash hfill}\\ +N_{z}L_{tr}\int_{d_{\text{0}}+7\overset{s}{\underset{epc}{\delta }}}^{\infty }\begin{array}{c} 2L_{c}{\delta_{c}}^{\frac{\text{1}}{\text{2}}}{\left\{2R_{c}-\left(z-d_{\text{0}}\right)\right\}}^{\frac{\text{1}}{\text{2}}}\\ \cdot \sigma^{-1}{\left(2\pi \right)}^{-1/2}exp\left\{-z^{\text{2}}/\left(2\sigma^{\text{2}}\right)\right\}dz \end{array}\mathrm{\backslash hfill} \end{array}$$ (19)

When contact deformation of asperities is in the range of $\delta_{\text{c}}\leq \delta_{\text{ep}}$, asperities will experience the first stage of deformation. According to definition of stiffness, the partial derivative of Eq. (6) is calculated, thus getting expression of contact stiffness $\;\overset{\text{s}}{\underset{\text{ci}\_\text{e}}{k}}$ in the first deformation stage of asperities:


$$\overset{s}{\underset{e}{k}}\left(\delta_{c}\right)=\frac{\partial \overset{s}{\underset{e}{f}}\left(\delta_{c}\right)}{\partial \delta_{c}}\text{=}\frac{0.08\pi E^{*}\left(R_{c}-\delta_{c}\right)L_{c}}{R_{c}}$$ (20)

In this range, $\delta_{\text{c}}\geq \overset{s}{\underset{\text{ep}}{\delta }}$ and asperities experience the second stage of deformation. The deformation properties in the second stage differ from those in the first stage, with plastic deformation dominating. Similarly, according to the definition of stiffness, the stiffness produced by the deformation of asperities in this stage can be obtained by calculating the derivative of deformation in this stage. In other words, the derivative of deformation is calculated according to Eq. (13). The contact stiffness expression for this deformation stage is:


$$\overset{s}{\underset{p}{k}}\left(\delta_{c}\right)=\frac{\partial \overset{s}{\underset{p}{f}}\left(\delta_{c}\right)}{\partial \delta_{c}}=\frac{\left\{{\left(\frac{\text{1}}{\text{2}}\right)}^{\text{2}}H_{\text{0}}L_{\text{ci}}{\left(R_{c}-\frac{\delta_{c}}{\text{2}}\right)}^{\frac{\text{1}}{\text{2}}}\right\}}{{\delta_{c}}^{\frac{\text{1}}{\text{2}}}}-\frac{\left\{{\left(\frac{\text{1}}{\text{2}}\right)}^{\text{2}}H_{\text{0}}L_{\text{ci}}{\delta_{c}}^{\frac{\text{1}}{\text{2}}}\right\}}{2{\left(R_{c}-\frac{\delta_{c}}{\text{2}}\right)}^{\frac{\text{1}}{\text{2}}}}$$ (21)

The total stiffness of interface contact is equal to the integral of the stiffness of each asperity along the height direction of the interface, including the stiffness of asperities in the first and second stages. The total stiffness is expressed as:


$$\begin{array}{c} \overset{jo}{\underset{t}{K}}\left(\delta_{c}\right)\text{=}N_{z}L_{tr}\int_{\text{0}}^{d_{\text{0}}+7\overset{s}{\underset{epc}{\delta }}}\overset{s}{\underset{e}{k}}\left(z-d_{\text{0}}\right)\cdot \varphi \left(z\right)dz\text{+}N_{z}L_{tr}\int_{d_{\text{0}}+7\overset{s}{\underset{epc}{\delta }}}^{\infty }\overset{s}{\underset{p}{k}}\left(z-d_{\text{0}}\right)\cdot \varphi \left(z\right)dz\mathrm{\backslash hfill}\\ =N_{z}L_{tr}\int_{\text{0}}^{d_{\text{0}}+7\overset{s}{\underset{epc}{\delta }}}\frac{0.08\pi E^{*}\left\{R_{c}-\left(z-d_{\text{0}}\right)\right\}L_{c}}{R_{c}}\cdot \sigma^{-1}{\left(2\pi \right)}^{-1/2}exp\left(-z^{\text{2}}/2\sigma^{\text{2}}\right)dz\mathrm{\backslash hfill}\\ +N_{z}L_{tr}\int_{d_{\text{0}}+7\overset{s}{\underset{epc}{\delta }}}^{\infty }\begin{array}{c} \frac{\left\{H_{\text{0}}L_{\text{ci}}{\left(\frac{\text{1}}{\text{2}}\right)}^{\text{2}}{\left(R_{c}-\frac{z-d_{\text{0}}}{\text{2}}\right)}^{\frac{\text{1}}{\text{2}}}\right\}}{{\delta_{c}}^{\frac{\text{1}}{\text{2}}}}-\frac{\left\{{\left(\frac{\text{1}}{\text{2}}\right)}^{\text{2}}H_{\text{0}}L_{\text{ci}}{\left(z-d_{\text{0}}\right)}^{\frac{\text{1}}{\text{2}}}\right\}}{2{\left(R_{c}-\frac{\delta_{c}}{\text{2}}\right)}^{\frac{\text{1}}{\text{2}}}}\\ \cdot \sigma^{-1}{\left(2\pi \right)}^{-1/2}exp\left(-z^{\text{2}}/2\sigma^{\text{2}}\right)dz \end{array}\mathrm{\backslash hfill} \end{array}$$ (22)

Gonzalez-Valadez et al. [42] reported through an experimental study in 2010 that the plastic deformation stage of interface asperities would be gradually eliminated after multiple applications of surface pressure. Under this circumstance, only the contact characteristics produced by the elastic deformation of interfacial asperities are considered. When only the elastic deformation of asperities is considered, the actual contact area, contact pressure, and contact stiffness of the interface can be expressed as follows:


$$\begin{array}{c} \overset{jo}{\underset{t}{A}}\left(\delta_{c}\right)=N_{z}L_{tr}\int_{d_{\text{0}}}^{+\infty }\overset{s}{\underset{ci\_e}{s}}\left(z-d_{\text{0}}\right)\cdot \varphi \left(z\right)dz\mathrm{\backslash hfill}\\ =N_{z}L_{tr}\int_{d_{\text{0}}}^{d+\infty }\begin{array}{c} 0.8{\left\{2R_{c}\left(z-d_{\text{0}}\right)-{\left(z-d_{\text{0}}\right)}^{\text{2}}\right\}}^{0.5}L_{c}\\ \cdot \sigma^{-1}{\left(2\pi \right)}^{-1/2}exp\left\{-z^{\text{2}}/\left(2\sigma^{\text{2}}\right)\right\}dz \end{array}\mathrm{\backslash hfill} \end{array}$$ (23)

$$\begin{array}{c} \overset{jo}{\underset{t}{F}}\left(\delta_{c}\right)\text{=}N_{z}L_{tr}\int_{\text{0}}^{+\infty }\overset{s}{\underset{e}{f}}\left(z-d_{\text{0}}\right)\cdot \varphi \left(z\right)dz\mathrm{\backslash hfill}\\ =N_{z}L_{tr}\int_{\text{0}}^{+\infty }\begin{array}{c} \frac{\text{1}}{25R_{c}}\pi E^{*}\left\{\begin{array}{c} @l@2R_{c}\left(z-d_{\text{0}}\right)\\ -{\left(z-d_{\text{0}}\right)}^{\text{2}} \end{array}\right\}L_{c}\\ \cdot \sigma^{-1}{\left(2\pi \right)}^{-1/2}exp\left\{-z^{\text{2}}/\left(2\sigma^{\text{2}}\right)\right\}dz \end{array}\mathrm{\backslash hfill} \end{array}$$ (24)

$$\begin{array}{c} \overset{jo}{\underset{t}{K}}\left(\delta_{c}\right)\text{=}N_{z}L_{tr}\int_{\text{0}}^{+\infty }\overset{s}{\underset{e}{k}}\left(z-d_{\text{0}}\right)\cdot \varphi \left(z\right)dz\mathrm{\backslash hfill}\\ =N_{z}L_{tr}\int_{\text{0}}^{+\infty }\frac{0.08\pi E^{*}\left\{R_{c}-\left(z-d_{\text{0}}\right)\right\}L_{c}}{R_{c}}\cdot \sigma^{-1}{\left(2\pi \right)}^{-1/2}exp\left(-z^{\text{2}}/2\sigma^{\text{2}}\right)dz\mathrm{\backslash hfill} \end{array}$$ (25)

## 3. Results and discussions

In this section, numerical parameters of fixed contact stiffness of rough surface are investigated. The interface separation is used as a variable to study influences of *σ/R*_c_ on contact force and contact stiffness of interfaces. Three steel samples with different surface parameters are applied to study influences of surface morphology on interfacial contact stiffness and contact area. Surface statistical parameters of different samples are listed in Table 1 (Nuri and Halling, 1975 [43]). Parameters are analyzed by using steel materials of *E*_1_ = *E*_2_ = 210GPa, *v*_1_ = *v*_2_ = 0.3, and *A*_n_ = 100mm^2^. The combined Young’s modulus of elastic solid is *E*_s_ = 115.4 GPa [44]. The yield strength is *S*_y_ = 500 MPa.

**Table 1:** Static parameters of different surface morphologies [43].

Samples	*σ*(μm)	*R*_c_(μm)	*n*(m^-2^)
1	0.16	16.81	2.310 × 10^10^
2	1.35	7.14	1.457 × 10^10^
3	3.94	6.12	9.960 × 10^9^

In this section, numerical parameters of the fixed contact stiffness of rough surfaces are investigated. The interface separation is used as a variable to study the influences of *σ*/*R*_c_ on the contact forces and contact stiffness of interfaces. Three steel samples with different surface parameters are used to study the influences of surface morphology on interfacial contact stiffness and contact area. The surface statistical parameters of different samples are listed in Table 1 (Nuri and Halling, 1975 [43]). Parameters are analyzed using steel materials with *E*₁ = *E*₂ = 210 GPa, *ν*₁ = *ν*₂ = 0.3, and *A*ₙ = 100 mm². The combined Young’s modulus of the elastic solids is *E*ₛ = 115.4 GPa [44]. The yield strength is *S*_y_ = 500 MPa.

Suppose the nominal contact area is *A*_n_ = 100 mm^2^. Statistical parameters of different surface morphologies are listed in Table 1.

**Fig 7:**
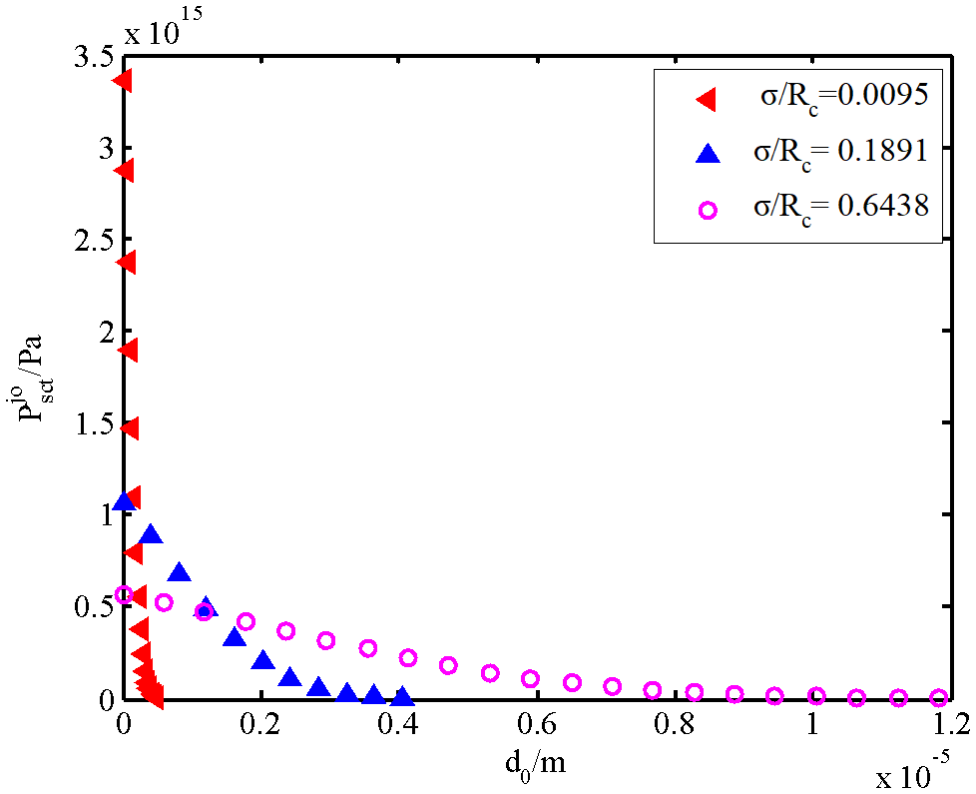
Interfacial contact forces.

Variations of the fixed interfacial contact force with interfacial separation are shown in Fig 7. A smaller *σ* results in a smaller interfacial separation required to produce contact force. A larger *σ* results in a larger interfacial separation at which contact force can be produced. With the reduction of interfacial separation, the interfacial contact force exhibits nonlinear growth.The higher *σ/R*_c_ value indicates the lower nonlinear growth rate of contact force. In other words, the curve is flatter. The lower *σ/R*_c_ value reflects the higher nonlinear growth rate of contact force, and the curve is sharper.

Moreover, when the interfacial separation is 0, the interfacial contact force is negatively related with *σ/R*_c_ value. This is because the asperity height distribution on interface is more dispersed if *σ* is higher. With the reduction of interfacial separation, more asperities enter into the plastic deformation. Hence, the final contact force is negatively related with *σ*.

**Fig 8:**
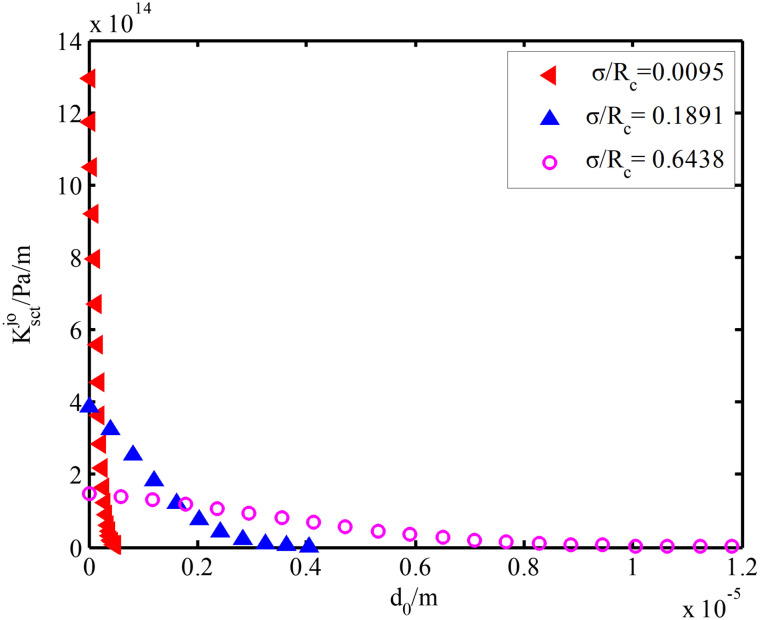
Interfacial contact stiffness.

Variations of fixed interfacial contact stiffness with interfacial separation are shown in Fig 8. Clearly, interfacial contact stiffness presents a nonlinear growth trend with the reduction of interfacial separation. The nonlinear growth rate of contact stiffness is lower when the *σ/R*_c_ ratio is higher, manifested by the smaller slope of the curve. On contrary, the nonlinear growth of contact stiffness is higher when the *σ/R*_c_ ratio is smaller, manifested by the larger slope of the curve. Besides, when interfacial separation is 0, interfacial contact stiffness is negatively related with *σ/R*_c_ value. This is because height distribution of interfacial asperity is more dispersed when *σ* is higher. More asperities enter into the plastic deformation with the reduction of interfacial separation. Therefore, the final contact stiffness is negatively related with *σ*.

## 4. Comparison and discussions

In this section, the contact stiffness results predicted by the proposed model in Eq. (22) are compared with measured results of Gonzalez-Valadez et al. [42].

Gonzalez-Valadez et al. [42] measured normal contact stiffness of rough surface contact through the spring model by using ultrasonic technology, as shown in Eq. (26):


$$\overset{s}{\underset{u}{K}}=\frac{Z\omega }{\text{2}}\sqrt{\frac{\text{1}}{{\left|R\right|}^{\text{2}}}-1}$$ (26)

where |R| is the modulus of ultrasonic reflectance. *Z* is the acoustic impedance of contact asperities. *ω* is the angular frequency of ultrasonic waves. Eq. (25) is a universal expression for stiffness calculation of the spring model.

The structural alloy steel is used as experimental materials. The equivalent elasticity modulus of materials is *E* = 114 GPa, Poisson’s ratio is *ν* = 0.29, and nominal contact area is *A*_n_ = 95 mm^2^. The roughness parameters of test samples are listed in Table 2. The contact stiffness values calculated by the developed acoustic model are also compared with predictions of two statistical contact models: pure-elasticity GW model and the elastic-plastic KE model.

**Table 2:** Experimental statistical parameters of rough surface samples [42].

Sample	σ (m)	*n*(m^-2^)	*β* (μm)
1 *R*_a_ = 1.58μm	2.04 × 10^−6^	7.324 × 10^9^	6.337
2 *R*_a_ = 2.42μm	3.10 × 10^−6^	6.209 × 10^9^	5.427
3 *R*_a_ = 3.09μm	3.90 × 10^−6^	5.513 × 10^9^	4.954

### 4.1 Effect of loading on roughness and stiffness

**Fig 9:**
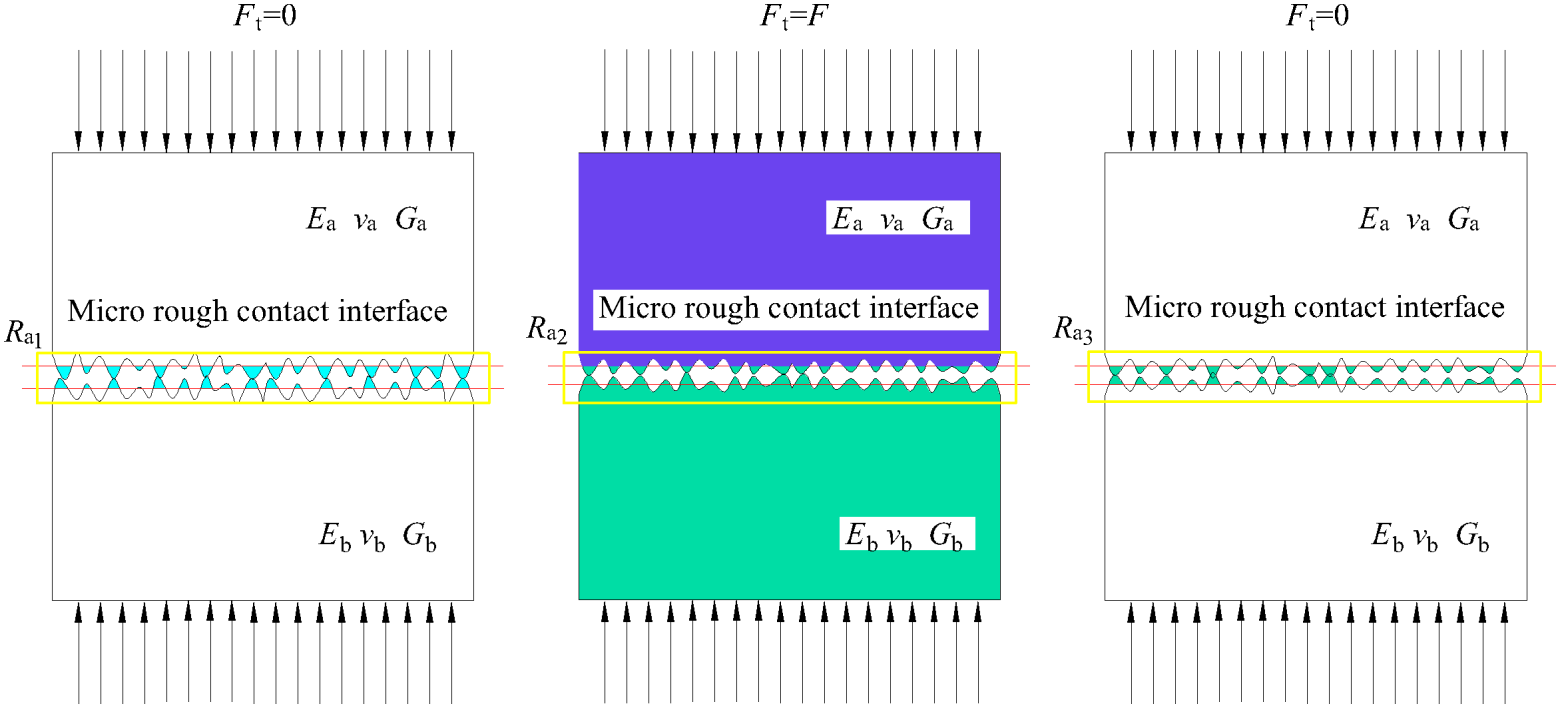
The impact of loading on the interface. **(a)** Before loading. **(b)** During loading. **(c)** After loading.

**Fig 10:**
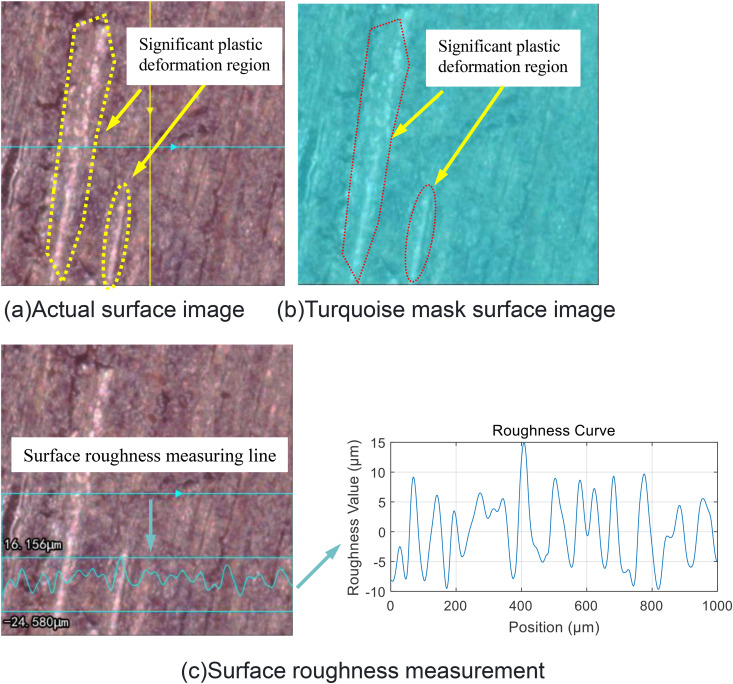
Detection of Surface 1 plastic deformation zones under compression. **(a)** Actual surface image. **(b)** Turquoise mask surface image. **(c)** Surface roughness measurement.

**Fig 11:**
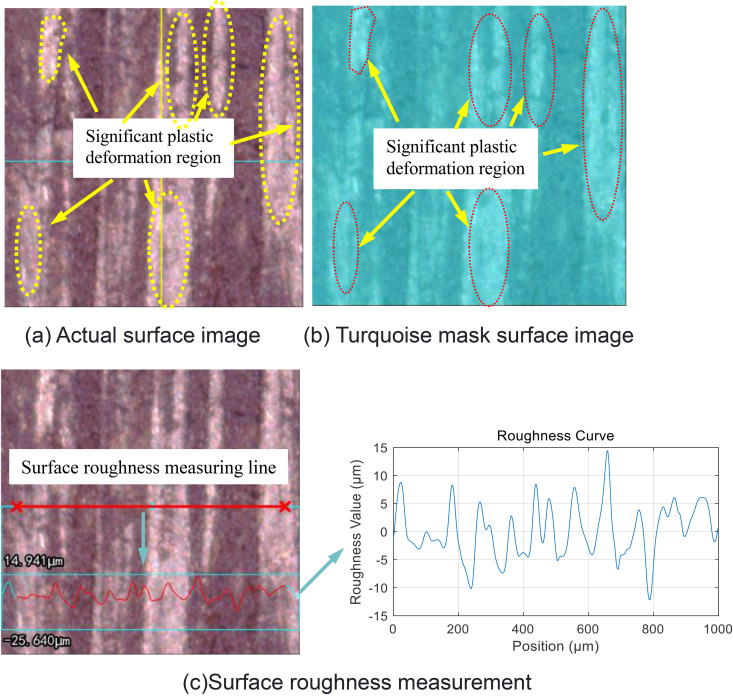
Detection of Surface 2 plastic deformation zones under compression. **(a)** Actual surface image **(b)** Turquoise mask surface image.

**Fig 12:**
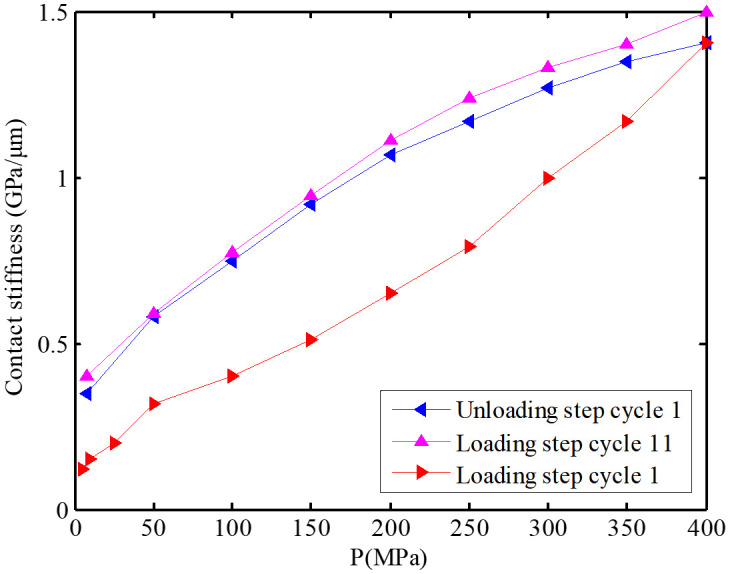
The influence of load on interface stiffness.

In the relevant experiments, when the researchers applied multiple loads to the joint surface, they found that the interface would change as shown in Fig 9 [42]. A remarkable phenomenon is that the surface roughness after loading is lower than the initial roughness, that is, *R*_a1_ > *R*_a3_. This phenomenon has triggered an in-depth exploration of the causes of the roughness change. In order to identify the root causes of the roughness change, this study carefully designed and carried out a series of joint surface loading experiments. Two different groups of joint surfaces were selected for the experiment, and a bolt pre-tightening force of 40 N·m was applied to each group, but the number of repetitions was different: one group was repeated 10 times, and the other group was repeated 3 times. This setting was intended to compare the effects of different numbers of loading cycles on the joint surface. Subsequently, a high-precision white light interferometer was used to detect the two groups of joint surfaces after loading, with a focus on observing the changes in the surface asperities. The test results are shown in Figs 10 and 11. From these figures, two distinct regions can be clearly distinguished: the darker region and the whitish region. Through further analysis and evaluation, it was determined that the whitish region corresponds to the plastic deformation region. The occurrence of this phenomenon is closely related to the plastic properties of the asperity material. When the asperities are subjected to a large pressure, especially in the areas where the pressure exceeds the yield limit of the material, irreversible plastic deformation will occur. During this process, the upper part of the higher asperities gradually flattens and spreads, filling the adjacent low-lying areas. Taking the surface shown in Fig 10 as an example, due to the repeated application of the bolt pre-tightening force 10 times, the plastic deformation in the asperities continuously accumulates and intensifies, resulting in more and more asperities being reshaped. The originally sharp and protruding asperities gradually become shorter and flatter, and the height difference between the surface peaks and valleys decreases. In contrast, for the surface shown in Fig 11, due to the fewer number of applications of the bolt pre-tightening force (only 3 times), the cumulative effect of plastic deformation is not obvious, and the plastic deformation region is relatively small. It can be seen that the plastic deformation of the asperities under the action of pressure is the main factor leading to the change in surface roughness. This indicates that under specific conditions of bolt pre-tightening, the microscopic morphology of the material surface will change significantly, and the roughness will continuously decrease. From a microscopic perspective, the surface of any material is not an ideal smooth plane, but is composed of many asperities with different heights and random distributions, which determine the initial surface roughness. In summary, the repeated action of the bolt pre-tightening force causes the asperities to undergo plastic deformation, thereby reducing the surface roughness of the material and changing the microscopic contact state of the surface. In addition, the experimental research also shows [42] that after multiple loadings, not only will the interface roughness change, but the contact stiffness will also change. M. Gonzalez Valadez et al. [42] carried out 10 complete cycles after the initial loading-unloading cycle to ensure the complete elimination of residual plasticity. The obtained contact stiffness change curve is shown in Fig 12. As can be seen from Fig 12, after multiple loading cycles, the plastic deformation is basically eliminated, and the interface exhibits an elastic-like behavior. At the same time, this figure also shows the changes in stiffness of the interface during the loading and unloading processes. For example, in Fig 10, numerous tiny asperities have undergone multiple extrusion deformations, resulting in plastic deformation in certain areas of the entire surface, a reduction in surface undulations, and a decrease in roughness. The same phenomenon also occurs in Fig 11, where plastic deformation leads to a reduction in surface undulations and a decrease in roughness. In general, it is precisely due to the repeated action of the bolt pre-tightening force that the tiny asperities undergo plastic deformation, thus reducing the surface roughness of the material and changing the microscopic contact state of the surface. This series of experimental results provides important theoretical and experimental bases for understanding the behavior of the joint surface under multiple loadings.

### 4.2 Statics contact models

#### 4.2.1 Greenwood and Williamson model (GW model).

Greenwood and Williamson proposed a statistical asperity model of contact between rough surfaces and smooth flat surfaces. This model is based on the elastic Hertz contact of rough surface contact and the fact that asperity height of rough surface obeys to Gaussian distribution. GW model [22] is based on the pure-elastic Hertz contact theory. It only considers elastic deformation of asperity, without considerations to elastic-plastic and plastic deformation of asperities. The normal contact loads and normal contact stiffness of the whole surface can be gained from following equations:


$$F_{ba}\left(d_{\text{0}}\right)=\frac{\text{4}}{\text{3}}\rho E^{*}\overset{\frac{\text{1}}{\text{2}}}{\underset{\text{ba}}{R}}A_{\text{0}}\int_{d_{\text{0}}}^{\infty }{\left(z-d_{\text{0}}\right)}^{\frac{\text{3}}{\text{2}}}\varphi \left(z\right)dz$$ (27)

$$K_{ba}(d_{\text{0}})=2^{E}\overset{\frac{\text{1}}{\text{2}}}{\underset{\text{ba}}{R}}\rho A_{\text{0}}\int_{d_{\text{0}}}^{\infty }{\left(z-d_{\text{0}}\right)}^{\frac{\text{1}}{\text{2}}}\varphi (z)dz$$ (28)

where *R*_ba_ is the spherical radius. *A*_0_ is the nominal contact area of interface. *ρ* expresses the asperity density of rough surface.

#### 4.2.2 Kogut and Etsion model (KE model).

Kogut and Etsion [25] gave expression between contact load and deformation of single asperity through a finite element analysis by considering elasticity, elastic-plastic and plastic contact behaviors. In KE model, the contact load of interface is equal to the sum of loads produced by asperity elastic deformation in the interface, loads produced by elastic-plastic deformation and loads produced by plastic deformation. According to loads produced in different stages of elastic-plastic deformation of a single asperity proposed by Kogut et al. as well as loads produced by elastic and plastic deformation contact of a single asperity, the contact load of interface can be expressed as follows:


$$F_{ba}\left(d_{\text{0}}\right)=\rho A_{\text{0}}\left\{\begin{array}{c} @l@\int_{d_{\text{0}}}^{d_{\text{0}}+\delta_{e\_ep1}}\frac{\text{4}}{\text{3}}E^{*}\overset{\frac{\text{1}}{\text{2}}}{\underset{\text{ba}}{R}}{\left(z-d_{\text{0}}\right)}^{\frac{\text{3}}{\text{2}}}\varphi \left(z\right)dz\\ +\int_{d_{\text{0}}+\delta_{e\_ep1}}^{d_{\text{0}}+\delta_{e\_ep2}}\text{0.6867}C_{k}H_{\text{0}}\pi R_{ba}\delta_{e\_ep}{\left(\frac{z-d_{\text{0}}}{\delta_{e\_ep}}\right)}^{1.425}\varphi \left(z\right)dz\\ \text{+}\int_{d_{\text{0}}+\delta_{e\_ep2}}^{d_{\text{0}}+\delta_{e\_pc}}\text{0.9333}C_{k}H_{\text{0}}\pi R_{ba}\delta_{e\_ep}{\left(\frac{z-d_{\text{0}}}{\delta_{e\_ep}}\right)}^{1.263}\varphi \left(z\right)dz\\ +\int_{d_{\text{0}}+\delta_{e\_pc}}^{\infty }2\pi H_{\text{0}}R_{ba}\delta_{e\_ep}\varphi \left(z\right)dz \end{array}\right\}$$ (29)

where *C*_k_ is a coefficient of hardness; *H* = 2.8*S*_y_, which is related with Poisson’s ratio (*v*) of materials. Besides, *q* = 0.454 + 0.41*v* [18].


$$\delta_{e\_ep1}={\left(\frac{\pi C_{k}H_{\text{0}}}{2E}\right)}^{\text{2}}R_{ba}$$ (30)

$$\delta_{e\_ep2}=6{\left(\frac{\pi C_{k}H_{\text{0}}}{2E}\right)}^{\text{2}}R_{ba}$$ (31)

$$\delta_{e\_pc}=110{\left(\frac{\pi C_{k}H_{\text{0}}}{2E}\right)}^{\text{2}}R_{ba}$$ (32)

*R*_ba_ is a spherical radius. *A*_0_ is a nominal contact area of interface. *ρ* is density of asperity of rough surface.

The expression of contact stiffness of interface is:


$$K_{ba}\left(d_{\text{0}}\right)=\rho A_{\text{0}}\left\{\begin{array}{c} @l@\int_{d_{\text{0}}}^{d_{\text{0}}+\delta_{e\_ep1}}2E^{*}\overset{\frac{\text{1}}{\text{2}}}{\underset{\text{ba}}{R}}{\left(z-d_{\text{0}}\right)}^{\frac{\text{1}}{\text{2}}}\varphi \left(z\right)dz\\ +\int_{d_{\text{0}}+\delta_{e\_ep1}}^{d_{\text{0}}+\delta_{e\_ep2}}\;\frac{\text{2}}{\text{3}}\times 1.03\times 1.425\pi R_{ba}C_{k}H_{\text{0}}{\left(\frac{z-d_{\text{0}}}{\delta_{e\_ep}}\right)}^{0.425}\varphi \left(z\right)dz\\ +\int_{d_{\text{0}}+\delta_{e\_ep2}}^{d_{\text{0}}+\delta_{e\_pc}}\;\frac{\text{2}}{\text{3}}\times \text{1.4}\times \text{1.263}\pi R_{ba}C_{k}H_{\text{0}}{\left(\frac{z-d_{\text{0}}}{\delta_{e\_ep}}\right)}^{0.263}\varphi \left(z\right)dz\\ +\int_{d_{\text{0}}+\delta_{e\_pc}}^{\infty }2\pi H_{\text{0}}R_{ba}\varphi \left(z\right)dz \end{array}\right\}$$ (33)

### 4.3 Results and discussions

**Fig 13:**
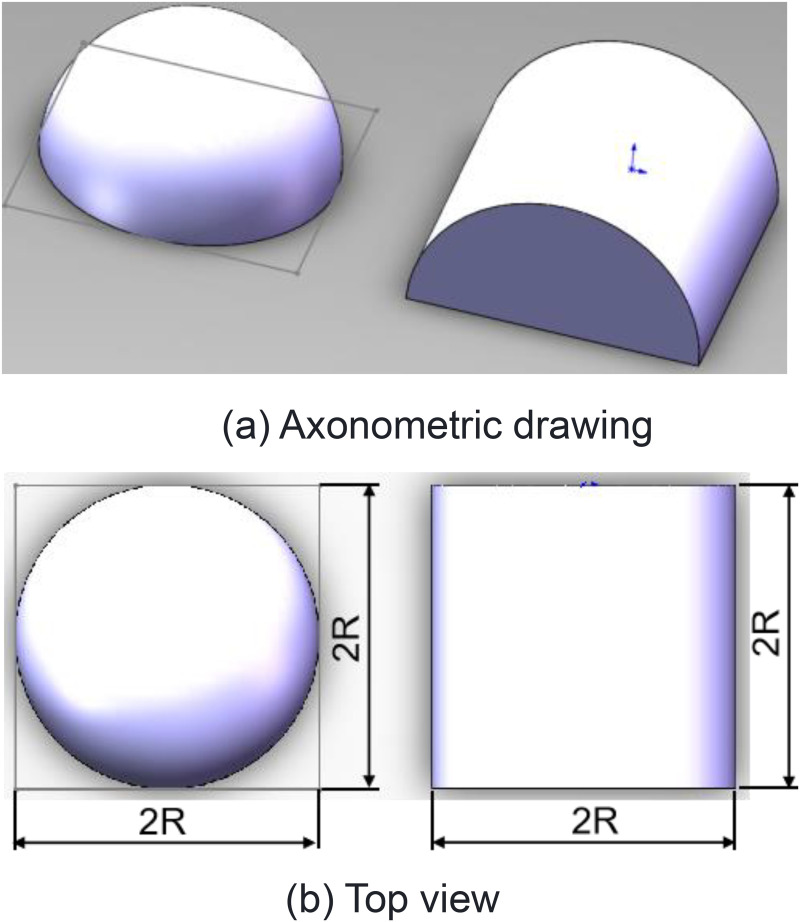
Equivalence of sphere and cylinder. **(a)** Axonometric(al) drawing **(b)** Top view.

It can be seen from expressions of interfacial contact stiffness in Eq. (28) and Eq. (33), *σ*, *H*_0_, *ρ* and *R*_ba_ are all essential conditions to determine interfacial contact stiffness per unit area in the statistical contact model. It can be seen from Eq. (22) that interfacial contact stiffness per unit area in the model is related with *R*_c_, *σ*, and *N*_z_. Since the proposed model is a cylindrical contact model that has significant differences from spherical contact model, the cylindrical contact model is equivalent to a spherical contact model.Results are shown in Fig 13. On this basis, validity of the proposed model is verified through experiments.

**Fig 14:**
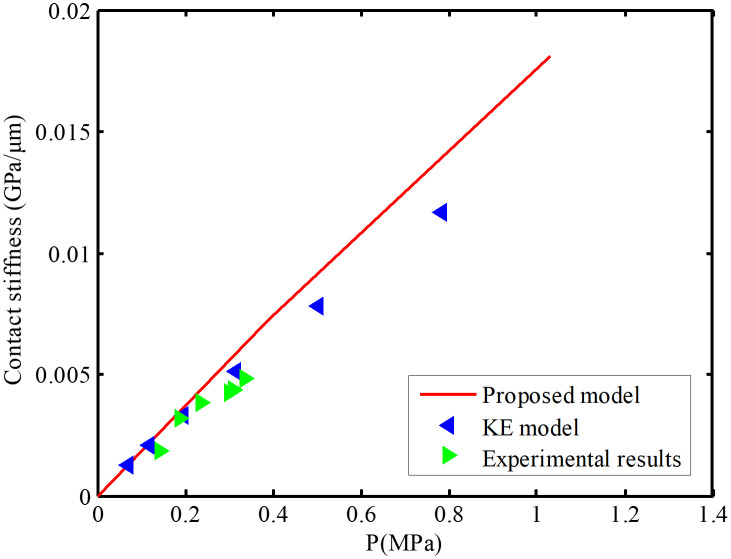
Comparison of experimental contact stiffness of interface after first loading-unloading cycles with results of the proposed model and KE model (*R*_a_ = 0.151 μm, *σ* = 0.189μm).

For the rough interface, the predicted values of normal contact stiffness are compared with experimental data [11]. Parameters of experimental samples, including nominal contact area *A*_n_ = 1.824 mm^2^, equivalent modulus of elasticity *E*_s_ = 105.3 GPa, Poisson’s ratio *v* = 0.29, standard deviation *σ* = 0.189 *μm* (Given *σ* = 0.189 μm, according to the formula $R_{\text{a}}={\sigma /\left(\pi /2\right)}^{0.5}$, *R*_a_ = 0.151 μm), *R*_*ba*_ = 2.402 μm, and *N* = 0.1256 μm^2^, are used for calculation. As shown in Fig 14, the reason for the difference between the predicted values and the experimental results may be attributed to the non linearity of the rough interface in the experiment.

In order to verify the validity of the model on a larger scale, the following work has been done. In many published theoretical and experimental results concerning normal contact stiffness of rough surface, surface roughness value (*σ*) of the test samples has been reported. The distribution density *ρ* and the radius *R*_ba_ of the asperity are also reported. Xiao Huifang found that on one hand, Gonzalez – Valadez et al. (2010) provided experimental results of normal contact stiffness of the contact interface. On the other hand, they proposed parameters of all studying samples to calculate statistical parameters *n* and *R* which are needed to calculate contact stiffness. The calculated results are listed in Table 3.

**Table 3:** Experimental statistical parameters of equivalent asperity rough surface samples to the study of Gonzalez-Valadez et al.

Sample	σ (m)	*n*(m^-2^)	*R*_ci_ (μm)	*L*_ci_
1 *R*_a_ = 1.58μm	2.04 × 10^−6^	7.324 × 10^9^	6.337	2 × 6.337
2 *R*_a_ = 2.42μm	3.10 × 10^−6^	6.209 × 10^9^	5.427	2 × 5.427
3 *R*_a_ = 3.09μm	3.90 × 10^−6^	5.513 × 10^9^	4.954	2 × 4.954

**Fig 15:**
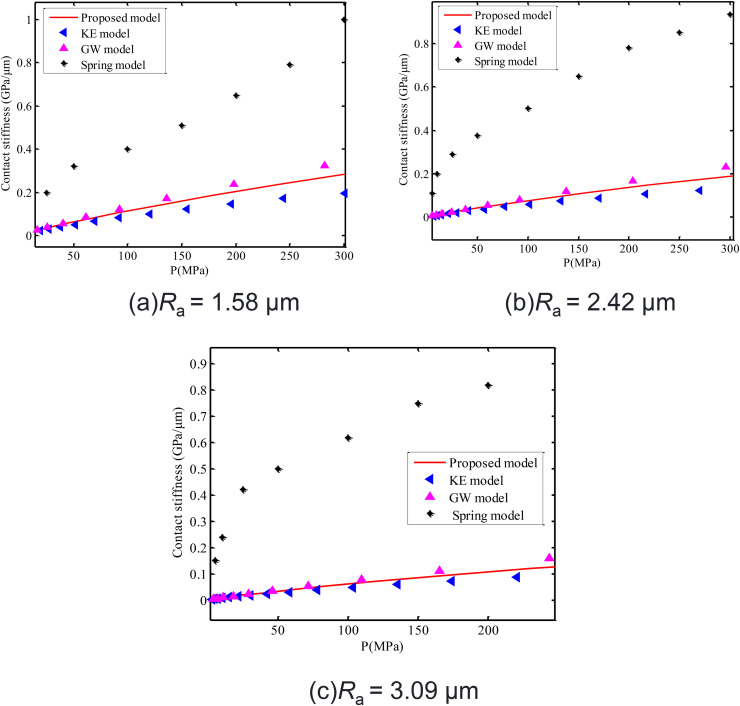
Comparison of experimental contact stiffness (spring model) of interface after 11 loading-unloading cycles with results of the proposed model, GW model and KE model. **(a)** *R*_a_ = 1.58 μm. **(b)** *R*_a_ = 2.42 μm. **(c)** *R*_a_ = 3.09 μm.

The spring experimental results (spring model) of Gonzalez-Valadez et al. [42], calculated stiffness of the proposed model, calculation results of KE model and GW model are shown in Fig 15. The calculated stiffness of the proposed model is very close to calculation results of GW statistical model. The experimental results of contact stiffness (spring model) are far higher than the calculated results of the proposed model, KE model and GW model. This is caused by ultrasonic attenuation of the spring model [44]. The calculated stiffness of the proposed model is very close to that of GW model, because both calculate stiffness from elastic deformation of asperities on interface. Such situation is corresponding to interfacial contact stiffness after practical multiple stress loading-unloading cycles of interfaces.

**Fig 16:**
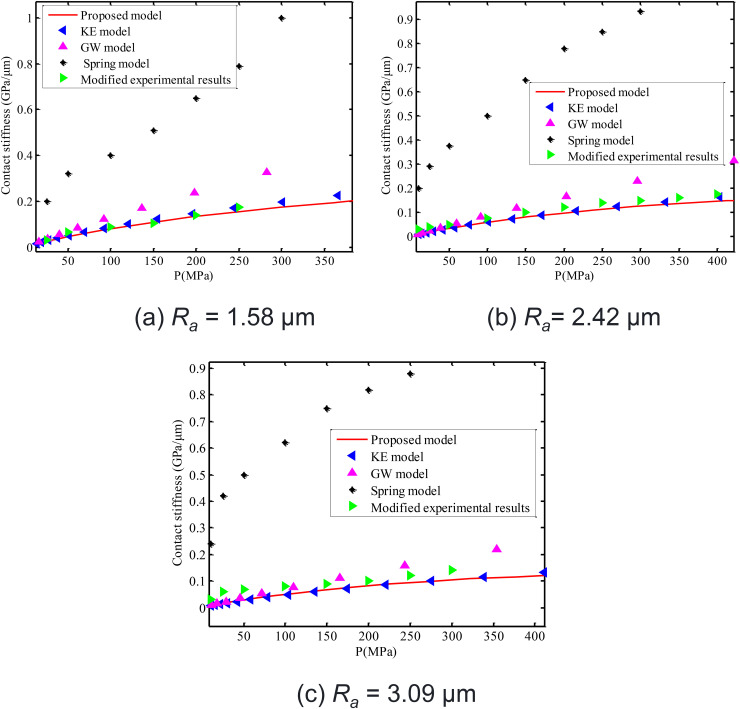
Comparison of experimental contact stiffness of interface after the first loading-unloading cycle (spring model) with results of the proposed model, GW model, spring model and experimental model corrected by Xiao Huifang. **(a)** *R*_a_ = 1.58 μm **(b)** *R*_a_ = 2.42 μm **(c)** *R*_a_ = 3.09 μm.

Calculated results of the proposed model, model corrected by Xiao Huifang, KE model, GW model and spring model of Gonzalez-Valadez et al. [42] are shown in Fig 16. Obviously, the calculated stiffness of the proposed model is very close to that of model corrected by Xiao Huifang. It is also very close to prediction results of KE model. This verifies reasonability of calculated results of the proposed model. Since influences of ultrasonic attenuation [44] are ignored, the measurement results of contact stiffness in the spring model are far higher than the calculated stiffness of the proposed model, KE model and GW model.

## 5. Conclusion

This study systematically explores the interface contact characteristics from the perspective of asperity morphology, with key contributions and innovations as follows:

(1) Development of novel statistical contact models tailored to asperity morphology

Based on the statistical distribution characteristics of rough interface asperities, two innovative calculation models are proposed: a statistical elastic contact model and a statistical elastic-plastic contact model. Unlike traditional models that oversimplify asperity features, these models explicitly integrate observed asperity morphology (captured via optical microscopy) and interface microstructure, establishing a direct link between microscopic asperity characteristics and macroscopic contact behavior. This addresses the gap in existing models where asperity morphology is often idealized, enhancing the physical realism of contact calculations.

(2) Targeted solution for contact stiffness calculation under specific loading conditions

The proposed models are uniquely designed to calculate two critical scenarios: (i) interface contact stiffness after plastic deformation is eliminated via multiple loading cycles, and (ii) contact stiffness during elastic-plastic deformation. This specificity fills the void in existing literature, which rarely distinguishes between these two states, enabling more accurate predictions for engineering applications involving repeated loading (e.g., bolted joints, mechanical interfaces under cyclic loads).

(3) Clarification of key influencing factors and validation of model robustness

This study quantitatively confirms that surface roughness of contact interfaces and material properties of contact bodies are dominant factors governing interface contact characteristics, providing a clear direction for optimizing interface performance in engineering practice. Moreover, rigorous validation-via comparative analysis with the original spring model, Xiao Huifang’s corrected contact model, and classical statistical contact models—demonstrates that the proposed models yield higher accuracy and broader applicability, verifying their reliability for practical engineering calculations.

In summary, this work advances the theoretical framework of interface contact mechanics by integrating microscopic asperity observation with statistical modeling, offering novel tools for predicting contact stiffness under complex loading conditions. The findings not only enrich the body of knowledge on rough surface contact but also provide actionable guidance for improving the design and performance of mechanical interfaces in industrial applications.
